# Differentiating early stage florid osseous dysplasia from periapical endodontic lesions: a radiological-based diagnostic algorithm

**DOI:** 10.1186/s12903-017-0455-5

**Published:** 2017-12-28

**Authors:** Victor Daviet-Noual, Anne-Laure Ejeil, Charles Gossiome, Nathan Moreau, Benjamin Salmon

**Affiliations:** 1 Dental Medicine Department, Bretonneau Hospital, HUPNVS, AP-HP, Paris, France; 2 Laboratory of Orofacial Neurobiology - Paris Diderot University, Paris, France; 3 EA2496 - Orofacial Pathologies Imaging and Biotherapies Lab, Dental School - Paris Descartes University Sorbonne Paris Cité, Montrouge, France

**Keywords:** Florid osseous dysplasia, Florid cemento-osseous dysplasia, Differential diagnosis, Periapical endodontic lesions, Dental pulp sensitivity test, Cone-beam CT

## Abstract

**Background:**

Osseous dysplasia (OD) is the most common fibro-osseous lesion of the jaw affecting the periapical region. Early stages of OD can resemble periapical radiolucencies, thus mimicking the radiological aspects of an endodontic pathology. Such radiolucent lesions affecting previously decayed or treated teeth are even more complex to interpret.

**Case presentation:**

The aim of this paper is to report a case-series of representative clinical situations describing the radiological features and illustrating the diagnostic workup of patients with florid osseous dysplasia (FOD). Emphasis is given to the endodontic implications of such periapical bone disease and the complexity of accurate diagnosis in the context of endodontic retreatment. We then propose a practical radiological-based diagnostic algorithm to assist the clinician in the diagnostic of OD periapical lesions.

**Conclusion:**

Periapical lesions may be confused with bone diseases such as osseous dysplasia, especially in the radiolucent initial stage. Knowledge of clinical features associated with a careful reading of cone beam CT images, such as fine opacities within the hypodense periapical lesion, may help determine the right diagnostic.

## Background

Osseous Dysplasia (OD) is the most common fibro-osseous lesion of the jaws affecting all parts of the maxilla and mandible, including the periapical region. OD lesions are classified as benign odontogenic neoplasms of the jaws with other fibro-osseous lesions in the 2005 World Health Organization classification, updated in 2017 [1]. Whereas historically these lesions were believed to be cemento-osseous, in 2001 Brannon and Fowler [2] showed in an histopathological study that these lesions were composed of abnormal bone and not a form of cementum. Therefore, since the 2005 classification, the term “cemento-osseous” should not be used anymore [3].

Different clinico-radiographic presentations are described, depending on whether the lesions are isolated or affect multiple anatomical areas and their specific location: focal, periapical, florid and a specific rare variant, the familial gigantiform cementoma [4, 5].

Florid osseous dysplasia (FOD) is the most extensive form with bony lesions involving multiple quadrants of the jaws. The etiopathogenesis of FOD is unknown but could derive from the periodontal ligament, the medullar bone or both [5]. Within these tissues and their immediate environment, fibrous tissue and metaplastic bone replace normal bone. Histological analysis of FOD reveals a cellular fibrous connective tissue containing new bone, osteoid and cementoid avascular material, accumulating over time. Radiographically, the appearance of these lesions matures over time, becoming increasingly radiopaque; three radiographic stages are thus described [4–6]:

- Stage 1 – osteolytic stage: radiolucent lesions,
- Stage 2 – mixed stage: radiolucent and radiopaque lesions,
- Stage 3 – osteogenic stage: radiopaque lesions.

As most FOD lesions are asymptomatic, diagnosis of such disease is based on incidental radiographic findings, usually during a routine dental exam, especially in middle-aged black female patients.

Diagnosis of FOD is based on the typical radiographic appearance assessed with standard two-dimensional projection radiographs (intraoral or panoramic radiography). Biopsy of such lesions is not recommended in typical presentations because of the risk of significant post-operative infection, probably secondary to the reduced vascular supply in these lesions [7].

In atypical cases involving the periapical region, three-dimensional radiographic assessment via cone-beam CT (CBCT) may be mandatory, especially in the early stages of disease, to facilitate differential diagnosis with other radiolucent periapical pathoses. Indeed, in the early radiolucent stage, such FOD lesions can mimic apical periodontitis, often leading to unnecessary endodontic treatment of healthy teeth and secondary infection of the underlying hypovascular bone. Diagnosis becomes even more challenging when the periapical radiolucent FOD lesion is associated with an endodontically-treated tooth.

The aim of this paper is to present a clinical case-series illustrating the radiological features of FOD involving the periapical region. Knowledge of such radiological features is essential for the differential diagnosis of apical periodontitis, especially on previously-treated teeth. Based on this illustrative case series and the authors’ experience of FOD, a practical radiological-based diagnostic algorithm is proposed to aid in the diagnosis of such perplexing radiographic presentations, a common clinical conundrum in routine endodontic practice.

## Cases presentation

We report three representative clinical cases of incidental discovery of florid osseous dysplasia, illustrating the different radiographic stages of the disease, the associated pathological consequences and finally the complexity of accurate diagnosis of periapical FOD in previously endodontically-treated teeth.

### Case #1 (Fig. 1)

#### Initial presentation and diagnosis of FOD

**Fig. 1:**
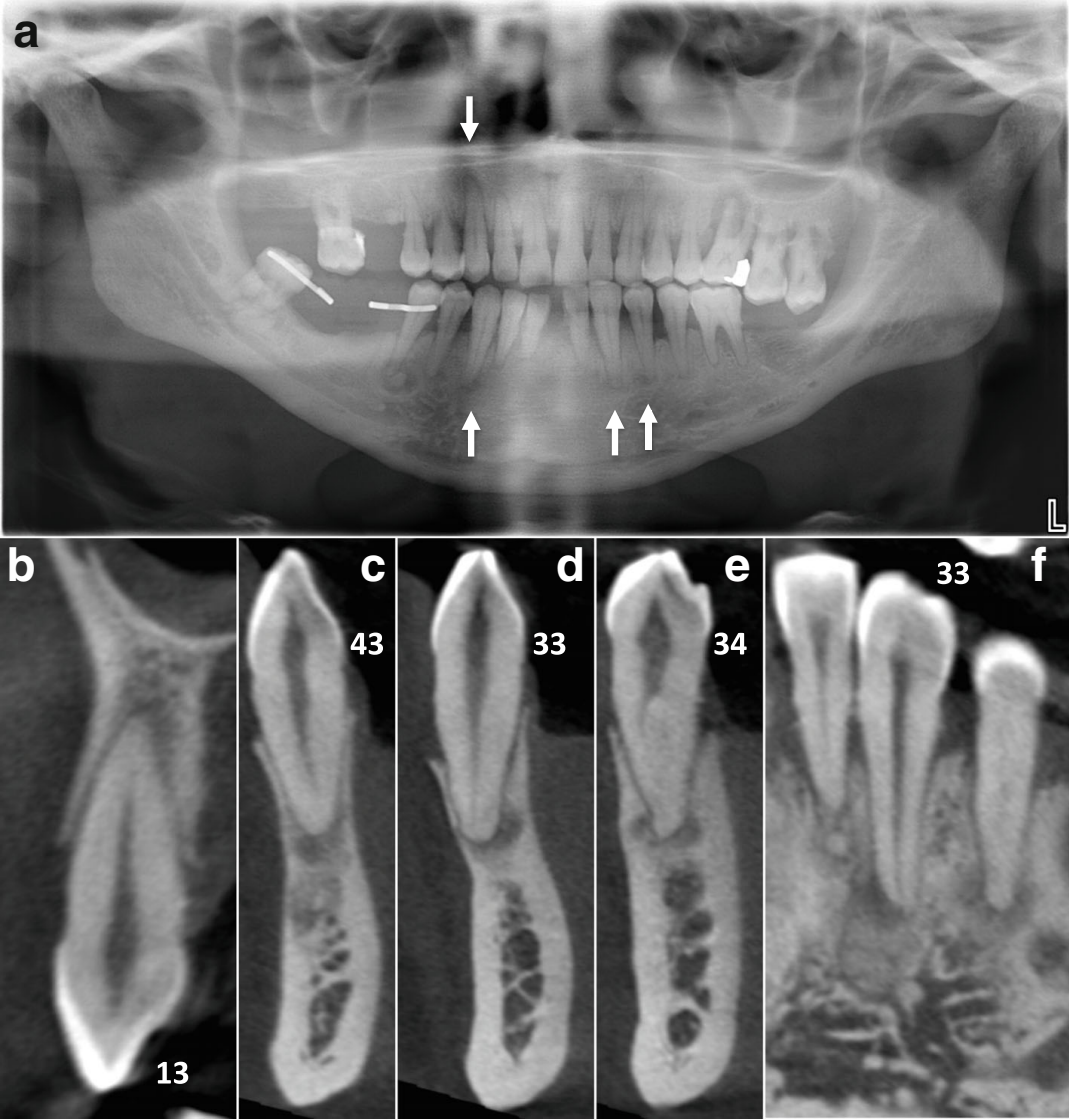
Stage 1 FOD with periapical involvement. Multiple periapical radiolucent lesions (white arrows) on vital teeth mimicking periapical endodontic lesions are observed on panoramic radiography (**a**) and detailed by CBCT cross sections focused on 13 (**b**), 43 (**c**), 33 (**d**), and 34 (**e** and **f**)

A 64 year-old African male patient, without any significant medical history, consulted the dental emergency department of the Bretonneau Hospital in Paris, with a mobile and painful upper left first molar resulting from a periodontal abscess. Aside from moderate to severe generalized alveolar bone loss, preoperative dental panoramic radiography revealed multiple periapical radiolucencies especially within the mandibular body (Fig. 1). Considering the radiological presentation typical of Florid Osseous Dysplasia, no biopsy was deemed necessary to confirm the clinical diagnosis.

#### Management of dental pathologies in the context of FOD

Extraction of the upper left first molar was performed under antibiotic prophylaxis (amoxicillin) and the patient was referred for a full periodontal treatment. At the one-week follow-up, the healing was uneventful.

#### Endodontic implications of FOD

Upon further medical interview, the patient did not report any history of trauma or swelling and all the teeth were vital except the upper left first molar. Thus, endodontic treatment in such clinical context is certainly not justified.

### Case #2 (Fig. 2)

#### Initial presentation and diagnosis of FOD

**Fig. 2:**
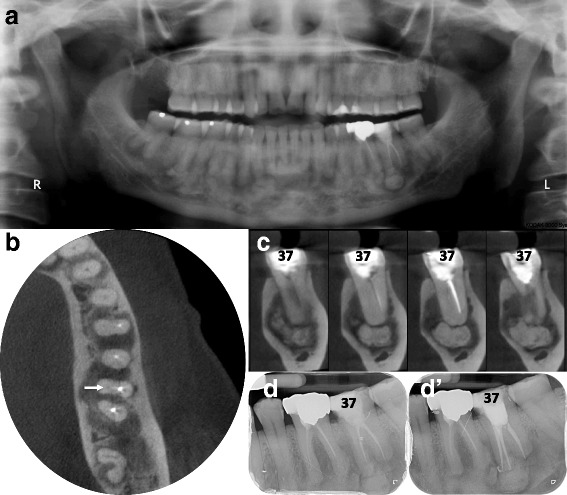
Stage 2 FOD with concomitant endodontic periapical lesion on symptomatic tooth 37. Diffuse mixed (radiolucent and radiopaque) periapical lesions are depicted on panoramic radiography (**a**). High-resolution/small field of view cone-beam CT allows axial (**b**) and bucco-lingual cross-sections (**c**) highlighting the lack of endodontic treatments and unobturated mesio-lingual canal (*white arrows*) associated with voluminous peri-radicular mixed-density images. (**d**, **d’**) Following endodontic retreatment tooth 37 became asymptomatic but the 6 months post-operative intra-oral x-ray (**d’**) was unable to show usual signs of alveolar bone healing as compared to the preoperative x-ray (**d**) because of superimposed FOD lesions

A 50 year-old African female patient, without any significant medical history, consulted the dental emergency department of the Bretonneau Hospital in Paris, with chronic pain and tenderness on the lower left second molar. Clinical and radiographic examinations (periapical radiograph [Fig. 2d]) revealed a previously endodontically-treated tooth, with a mixed-density periapical lesion composed of a round periapical radiopaque lesion of approximately 7 mm in diameter centered on the mesial root within a globally radiolucent periapical lesion including both roots of the tooth. Similar lesions were observed in the whole mandible on panoramic radiography (Fig. 2a). Small field of view (5x5cm) CBCT imaging confirmed the mixed density of the periapical pathology on the lower left second molar and allowed better visualization of the mesial periapical aspect confirming a globally radiopaque oval lesion surrounded by a radiolucent halo (Fig. 2c). Upon such radiographic presentation, a clinical diagnosis of Florid Osseous Dysplasia was made and no further investigations deemed necessary.

#### Management of dental pathologies in the context of FOD

High-resolution CBCT imaging of the lower left second molar also allowed to highlight the previous endodontic treatment failure of the lower left second molar, namely an untreated mesio-lingual canal. Treatment therefore consisted of an endodontic retreatment of the tooth, using mechanical Protaper® for root canal preparation followed by a vertical condensation obturation technique with heated gutta-percha. At the six-months follow-up, the patient was pain free and a ceramo-metallic crown was installed.

#### Endodontic implications of FOD

Although the patient could not recall the history of the lower left second molar and more specifically the reasons for the initial endodontic treatment, it is plausible that the initial consultation was made when the radiographic aspects of the FOD (stage 1 - radiolucent) could mimic periapical pathology, thus potentially leading to an endodontic overtreatment, especially on a tooth that was probably previously decayed (as suggested by the loss of the mesial marginal crest on the lower left second molar). This clinical case thus illustrates the difficulties of accurate diagnostic workup of periapical radiolucencies in previously treated and decayed teeth.

### Case #3 (Fig. 3)

#### Initial presentation and diagnosis of FOD

**Fig. 3:**
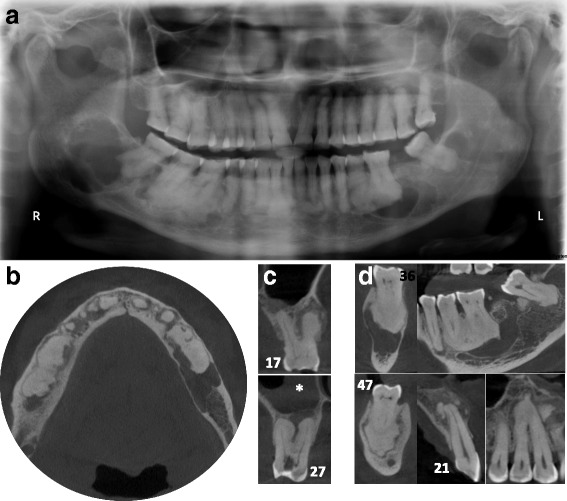
Stage 3 FOD presenting with multiple periapical radiopaque lesions associated with large and multiple osteolytic areas on panoramic radiography (**a**) and CBCT images (**b**-**d**). Histology confirmed simple bone cysts and bony/cementoid deposits compatible with giant florid osseous dysplasia. The Schneiderian membrane thickening (*white star*) associated with micro-perforations of the sinus floor adjacent to the periapical radiolucent lesion of tooth 27 confirms the endodontic origin of such lesion. In contrast, the lesion on tooth 17 appears of mixed-density, is limited to the alveolar bone and respects the sinus cavity (**c**)

A 72 year-old Cameroonian female patient, with a medical history of joint pain, gastric ulcer and hypertension, consulted the dental emergency department of the Bretonneau Hospital in Paris, with multiple tooth pain, more severe in the upper left maxillary region. After clinical and radiographic examinations, a diagnosis of symptomatic apical periodontitis on the upper left second molar was made and immediate root canal disinfection was undertaken. Orthopantomogram (Fig. 3a) and CBCT imaging (Fig. 3b, c) revealed multiple vast radiolucent and radiopaque lesions in the periapical regions of all mandibular molars (all teeth responding positively to pulp sensitivity tests), suggestive of FOD. These lesions were associated with several atypical multilocular radiolucent lesions in the left molar region and in both mandibular angles. Incisional biopsy was conducted on the right posterior mandibular lesion to ascertain the histological nature of such lesions. During the surgical procedure, a seemingly empty cavity was found following corticotomy and a sample of the overlying cystic membrane was harvested. Histopathological analysis of the tissue specimen revealed mild paucicellular fibrosis, with few lymphocytes, histiocytes, polymorphonuclear cells and numerous basophile structures compatible with bone or cement deposits. In sum, histopathological analysis was compatible with giant florid osseous dysplasia lesions, thus confirming the clinical diagnosis of FOD.

#### Management of dental pathologies in the context of FOD

Following initial diagnostic workup, the endodontic treatment of the upper left second molar was performed using a conventional technique without need of any specific premedication. The healing was uneventful.

#### Endodontic implications of FOD

In patients with advanced FOD lesions, diagnosis of such a condition is quite straightforward. Nevertheless, a doubt can still remain regarding teeth that present radiological aspects of OD but that also show significant tooth decay. Rigorous clinical exams including pulp sensitivity testing and periodontal probing as well as radiographic exams (2D and 3D imaging studies) are thus mandatory to allow precise diagnostic and suitable treatment of the periapical pathology. For instance, thickening of the Schneiderian membrane adjacent to periapical osteolytic image with micro-perforations of the sinus floor support the endodontic origin of the periapical radiolucency in the upper left second molar compared to the right side (Fig. 3C).

## Diagnosis and management of periapical FOD

**Fig. 4:**
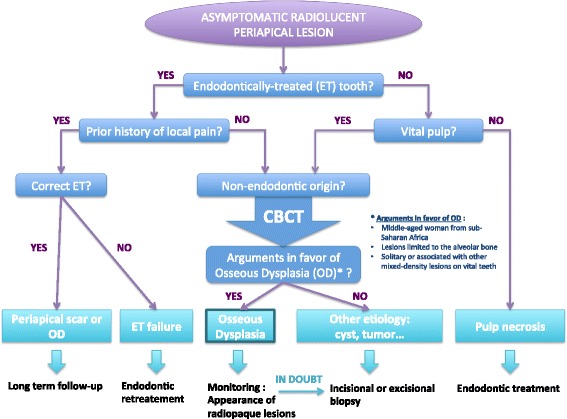
Radiological-based diagnostic algorithm to aid in differentiating stage 1 (osteolytic) periapical FOD from apical periodontitis

Diagnostic workup of unexplained radiolucent lesions must follow a strict methodology to evoke or rule out a possible FOD lesion. This methodology is summarized in Fig. 4.

First, clinical interview should focus on past history of dental pain, trauma, tooth decay or cracks. When faced with a previously endodontically-treated tooth, understanding the indication of such treatment is essential to determine if a possible misdiagnosis could have occurred. Indeed, spontaneous pulp necrosis in the absence of tooth decay, trauma or other local aggressive factors seems quite unlikely, especially in totally asymptomatic teeth. On the other hand, when faced with a periapical radiolucent lesion in an otherwise healthy tooth with a vital pulp, a non-endodontic origin of such lesion must be envisaged. Periapical osseous dysplasia should be considered in the following circumstances:

- Lesions occurring in middle-aged woman, especially those of sub-Saharan origin,
- Unique or multiple radiolucent, mixed or radiopaque lesions in the tooth bearing area, especially if occurring on vital teeth,
- Absence of symptomatology (in most of the cases).

If such arguments are present, a provisional diagnosis of osseous dysplasia can be provided. Management will consist in a close follow-up until these lesions develop the typical radiopaque appearance, confirming the diagnosis. To that extent, CBCT sections are of use in assessing the internal pattern of the lesion, with attention to minute microcalcifications, but also to rule out lesion expansion onto surrounding structures. In doubt, another origin must be evocated; typically odontogenic and non-odontogenic cysts or tumors of the jaws. A biopsy of such lesions is then required and treatment adapted to the underlying etiology.

## Discussion

Osseous dysplasia (OD), a rare and benign fibro-osseous lesion of the jaws, can mimic a radiolucent endodontic periapical lesion in the early stage of its maturation. Whereas differentiating such conditions in a vital tooth can be relatively straightforward, the situation becomes more complex when faced with endodontically-treated teeth, especially in cases of presumed inadequate treatments. Correct differential diagnosis is thus of paramount importance for the clinician, in order to avoid unnecessary iatrogenic dental treatments.

Florid osseous dysplasia (FOD) is the widespread form of osseous dysplasia; periapical OD is confined to the anterior mandible; focal OD occurs in a single sextant and familial gigantiform cementoma is an uncommon variant encountered preferentially in young patients that can lead to considerable bone expansion [8, 9].

In FOD, the molar and premolar teeth are the most frequently involved. FOD is usually asymptomatic and thus an incidental finding during a routine dental radiographic examination. The rare clinical symptoms observed are pain, swelling and local drainage but these are only encountered in cases of secondary infection, when the calcified masses are exposed in the oral cavity.

FOD follows an ethnic distribution, as shown in the systematic review of MacDonald-Jankowski in 2003: 59% of cases arise in African patients, 37% in Asians and 3% in Caucasians. In our patient series, collected within a French Parisian hospital, 92% of cases arose in African patients. Women are more affected than men and the disease occurring mostly around the fourth decade [10]. Several autosomal dominant cases of FOD have also been reported in the literature [5, 8]. Interestingly and similarly to the third case previously presented, simple bone cysts have been reported in association with FOD, but no conclusive explanation for such association has been found yet [11].

Because of its typical clinico-radiographic presentation, diagnosis of FOD is usually straightforward and does not require any additional investigations. For instance, the discovery of multiple periapical radiolucencies on vital teeth in different quadrants is a sufficient argument to evoke the diagnosis of Florid Osseous Dysplasia. There is no need for a biopsy in typical cases, especially considering the risk of secondary infection of these hypovascularized lesions. Nevertheless, when in doubt, a biopsy can be performed to rule out other fibro-osseous lesions or bone diseases [7].

Differential diagnosis of early stage FOD (Fig. 1) should include others periapical radiolucent lesions especially apical periodontitis, whereas radiopaque FOD lesions (Figs. 2 and 3) should be differentiated from chronic diffuse osteomyelitis, Paget disease and other fibro-osseous lesions such as ossifying fibroma (Fig. 6) and fibrous dysplasia [5, 7, 10, 12, 13]. In particular, differential diagnosis with ossifying fibroma can be difficult when faced with a small solitary lesion, both entities sharing near-identical radiological features.

As FOD is a benign usually self-limiting disease, treatment of such condition mostly consists of simple monitoring of the lesions, with an annual long-term clinical and radiographic follow-up [4, 5, 14, 15]. Patients should be informed of the slowly growing nature of FOD as well as the self-limiting behavior of the lesions. Complications of such disease are rare, mostly local surinfection of the lesions. In case of local FOD lesion infection, a surgical resection of the dysplastic fibro-osseous bone can be carried out under antibiotic treatment, taking into consideration the poor tissue diffusion of the antibiotic because of the avascular nature of these lesions. Symptomatic cases with swelling and deformation require a more complex management, usually a partial resection of the lesions to alleviate the symptoms. Several cases of osteomyelitis have been reported as a possible complication of FOD [16–18].

FOD is a condition of significant importance for the endodontist and general dental practitioner. Endodontic lesions and FOD may co-exist (Figs. 2 and 3) and FOD can affect the diagnosis, prognosis and monitoring of endodontic lesions [19]. For instance, when FOD lesions become radiopaque, the periodontal ligament space becomes invisible on radiographic examinations and can no more serve as an aid in determining accurate working length of the tooth. In cases where periapical surgery is required, clinicians must be aware of the risk of secondary infection of FOD lesions because of their avascular nature, strongly impairing periapical healing.

A classic clinical conundrum for the dental clinician is trying to accurately diagnose the radiolucent periapical lesion in an endodontically-treated tooth in a patient presenting with FOD. Several questions arise: Is the periapical lesion a FOD lesion, an apical periodontitis or a surinfection of a FOD lesion? Is the endodontic treatment a consequence of a misdiagnosed FOD lesion or an adequate treatment of a superinfected FOD lesion? Indeed, reports of lesions mimicking periapical endodontic lesions leading to inappropriate endodontic treatments are frequent in the literature [15].

**Fig. 5:**
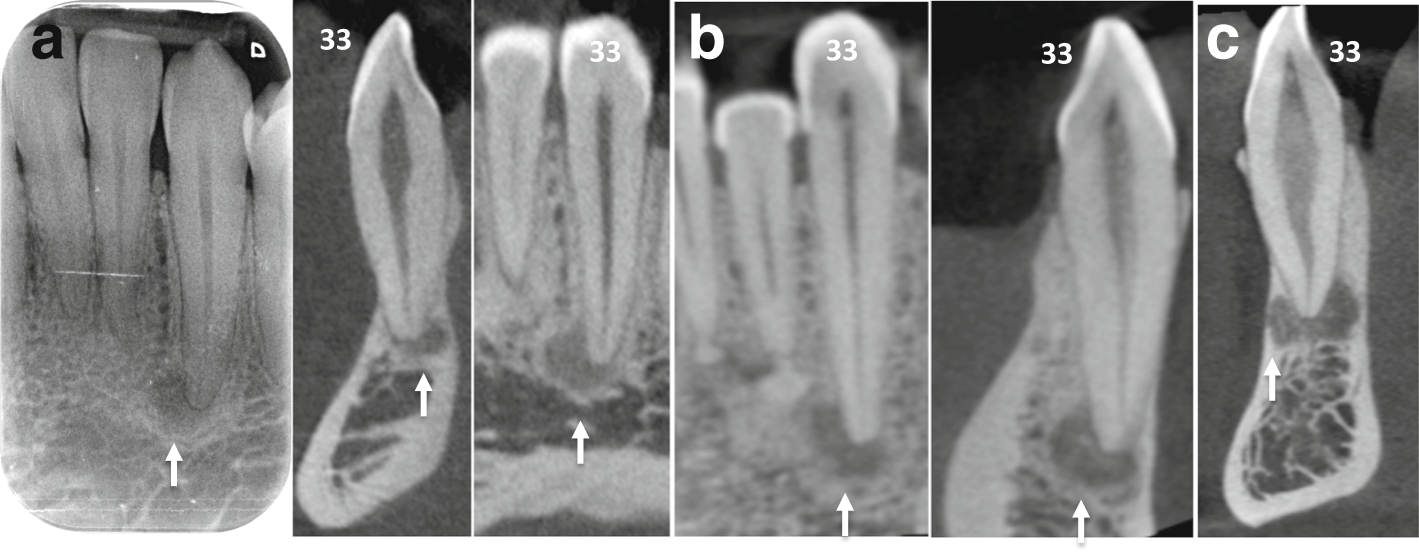
Additional examples of stage 1 OD mimicking endodontic periapical lesions. Careful examination of stage 1 OD depicted in CBCT might indicate faint differences as compared to endodontic lesions. For instance, the periapical OD radiolucency could appear slightly more dense compared to the surrounding trabecular space (*white arrows* in **a**) and/or can include discrete central hyperdense structures surrounded by a hypodense margin (*white arrows* in **b**). Moreover this complex is separated from the regular cancellous bone by a thicker hyperdense line. The whole process can protrude into the compact bone as well (*white arrows* in **c**)

**Fig. 6:**
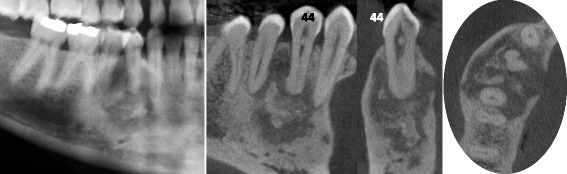
The radiological presentation of ossifying fibroma, a true neoplasm capable of significant growth, may be similar to the mixed radiolucent and radiopaque appearance of stages 2 or 3 FOD, but this disease tends to be more aggressive, causing bony expansion and occasional displacement of surrounding teeth

In this paper, we presented several clinical cases of patients presenting typical FOD lesions, in healthy teeth, endodontically-treated teeth or decayed teeth (Figs. 1, 2 and 3). As previously illustrated, diagnosis and management of such lesions can be quite challenging especially in stage 1 osteolytic FOD lesions, which can perfectly mimic apical periodontitis [20]. Whereas three-dimensional CBCT examination is of little use in typical FOD lesions (easily diagnosed on simple two-dimensional radiographs), it can be a significant aid in elucidating the nature of periapical pathology observed in FOD patients. Indeed, CBCT can provide useful information about the lesion’s limits, local extension and radiopacity [19]. For instance, thin cross-sections may underline faint differences regarding the radiodensity of stage 1 OD, which may seem slightly denser in contrast with the trabecular space or that can include minute micro-opacities (Fig. 5). Moreover, FOD lesions are self-limited above the mandibular canal (Fig. 3) and below the hard palate junction in the maxilla thus always limited to the alveolar process [3]. On the contrary, other fibro-osseous lesions such as ossifying fibroma (Fig. 6) show a more aggressive and expansive behavior, useful in differentiating the two conditions.

## Conclusion

Florid Osseous Dysplasia is a rare disease composed of slowly growing, quiescent fibro-osseous lesions presenting as radiolucent and/or radiopaque masses in the jaws and periapical region, which usually do not require any treatment except in case of complications.

Whereas typical radiopaque lesions are highly suggestive of the condition, early stage radiolucent periapical lesions can easily be confused with apical periodontitis especially in endodontically-treated teeth. Diagnosis and management of such confusing presentations must thus follow a rigorous methodology to avoid unnecessary iatrogenic dental treatments and possible disease-related complications.

## Abbreviations

CBCT: Cone-beam CT
FOD: Florid osseous dysplasia
OD: Osseous dysplasia
